# Awareness, knowledge, and beliefs about probiotics and prebiotics among Saudi adults: a cross-sectional study

**DOI:** 10.3389/fimmu.2024.1464622

**Published:** 2024-10-24

**Authors:** Areej Ali Alkhaldy

**Affiliations:** Department of Clinical Nutrition, Faculty of Applied Medical Sciences, King Abdulaziz University, Jeddah, Saudi Arabia

**Keywords:** probiotics, prebiotics, knowledge, awareness, beliefs

## Abstract

**Background:**

Probiotics and Prebiotics are essential for supporting both overall health and gastrointestinal health. However, the perception of these dietary components among the general public in Saudi Arabia is not well understood. The purpose of this study was to evaluate public awareness, knowledge, and beliefs regarding prebiotics and probiotics across Saudi Arabia.

**Materials and methods:**

Our cross-sectional study included 1,306 participants aged 18 years and above. Data were collected in Saudi Arabia between May and July 2023 using a self-administered online questionnaire via convenience sampling.

**Results:**

A high level of awareness was self-reported by only 21.9% of participants, whereas more than half (51.8%) of participants rated their level of awareness as low. Overall, 37.5% of participants displayed a high level of knowledge about probiotics and prebiotics, whereas 15.5% had a low level of knowledge. The majority of participants believed in the beneficial effects of probiotics and prebiotics on overall digestion/gut health (84.1%) and supporting the immune system (72.5%). However, less than half of participants believed in their beneficial effects on overweight/obesity (42.3%), stress management (35%), mental health/stress (29.2%), and heart health (28.7%).

**Conclusions:**

The obtained findings indicate sufficient levels of knowledge about prebiotics and probiotics among a population sample of Saudi adults. However, enhanced educational efforts and optimized strategies for promoting a comprehensive awareness and understanding of probiotics and prebiotics are recommended.

## 1 Introduction

Probiotic- and prebiotic-rich foods and supplements are attracting increasing interest owing to their potential health benefits (1, 2). The action mechanisms of probiotics include competitive elimination of pathogens, enhancement in the functions of the intestinal barrier, immunomodulation in the body host, and production of neurotransmitters (3, 4), while the action mechanisms of prebiotics included the inhibition of the damage of pathogen or immune system modulation, the enhancement of the function of the gut barrier, decreasing the pathogenic bacteria population, the production of short-chain fatty acids (SCFAs), and mineral bioavailability (5). Due to action mechanisms of both probiotics and prebiotics, they have been linked to a wide variety of health benefits, such as enhancing immune function, lowering blood cholesterol, preventing cancer, treating diarrhea associated with irritable bowel syndrome, improving lactose metabolism, and promoting the gastrointestinal microbiota (6–8).

In 2017, the global retail market for probiotic products was forecast to see a compound annual growth rate of 7%, while the size of the global prebiotic market was projected to increase by 12.7% between 2015 and 2025 (9, 10). In Saudi Arabia, the probiotics market is predicted to rise at a compound annual growth rate of 7.21% from US$ 0.0102 to 0.166 billion by 2027 (11). The Saudi Food and Drug Authority (SFDA) guideline of Data Requirements for Herbal & Health Products Submission state that probiotics products should be illustrated in Common Technical Document (CTD) format (for both over-the-counter and prescription). However, only the sections of quality modules of the CTD are mandatory (12). The purpose of this criterion is to ensure fulfilment of raw material and finished probiotic product quality standards. Moreover, the SFDA requires that probiotic product manufacturers adhere to the current Good Manufacturing Practices, similar to other regulatory authorities, and that they include the certificate with the product submission (13).

In 2020, Aldawsari et al. reported that with using a genotypic method, only one out of 22 probiotics products available in Saudi Arabia had confirmed the bacterial strain. The remaining of the 22 probiotics products showed different phenotypic methods. Yet, more than half of the studied probiotics products did not show the strain description on the labelling of the probiotic (13). In addition, Aldawsari et al. concluded their study by stating that the SFDA should adopt a new guideline to control and regulate probiotics. Recently, in May 2024, the SFDA updated the regulation of food supplements and issued a circular to define the classification criteria. Food supplements including prebiotics and probiotics will be classified as “Pharmaceutical Product” if intended to treat, prevent and/or diagnose a human disease (14).

Considering the significance of probiotics and prebiotics for various health outcomes, it is important to evaluate public understanding of the consumption of these food components. Informed consumers are better equipped to make dietary choices that promote health (15). By understanding the roles of probiotics and prebiotics in maintaining a healthy gut microbiota, individuals can make conscious decisions to incorporate foods rich in these components into their diet. Moreover, heightened awareness may foster greater demand for probiotic and prebiotic products, driving innovation in the food industry and facilitating access to these beneficial dietary components. Greater knowledge and understanding of probiotics and prebiotics among consumers are expected to empower individuals to take proactive steps toward managing their gastrointestinal health and improving their overall quality of life.

Nevertheless, previous research has revealed varying levels of understanding and awareness of prebiotics and probiotics among the general populations of different countries. A study conducted in Australia found that 58.9% of individuals were consuming probiotics and the consumption of probiotics was linked to the level of public awareness of prebiotic and probiotic terminology (16). Another study in Jordan showed that the understanding of probiotics and their potential applications among the general public was lacking (17). The authors suggested that more effort is needed to improve public awareness about the advantages of probiotics; for example, encouraging physicians and chemists to educate the public about probiotics could lead to greater consumer understanding (17). Guidance from general practitioners and other healthcare professionals may be essential in shaping public opinion and promoting probiotic intake. However, findings from previous surveys have suggested that the understanding of probiotics among healthcare practitioners is also mediocre, which may influence their likelihood of recommending probiotics (18, 19). Thus, focused educational campaigns targeting healthcare professionals are essential for improving public understanding of prebiotic and probiotic intake.

In Saudi Arabia, the public knowledge of probiotics and prebiotics is not well recognized, the majority of available studies focused on assessing the knowledge of probiotics and not prebiotics or synbiotics (13–16). Only one regional study conducted in Al-Qassim in 2019 assessed public understanding toward the knowledge, attitude, and perception about probiotics (20), while other works focused on evaluating knowledge, attitudes, and practice towards probiotics about probiotics among pediatricians in 2021 (21), health professionals in 2023 (22), and health care students in Riyadh in 2024 (23). The study performed in Al-Qassim indicated a limited level of knowledge about these nutritional components among the Saudi population, with only 26% of individuals familiar with probiotics (20).

Probiotics and prebiotics play integral roles in promoting gut health and overall well-being. Their definitions, common health benefits, and historical context provide a foundation for understanding their significance in modern dietary practices. In Saudi Arabia, the public understanding of these dietary substances is not well understood. However, studies in other countries have revealed gaps in public understanding and awareness of these dietary components, highlighting the important role of enhanced education and public awareness initiatives. Therefore, the aim of this study was to assess the public awareness, knowledge, and beliefs about probiotics and prebiotics across Saudi Arabia.

## 2 Materials and methods

### 2.1 Study design

In this cross-sectional study, participants were recruited in Saudi Arabia between May and July 2023 and asked to complete an online questionnaire. This study was approved by the Unit of the Biomedical Ethics Research Committee at King Abdulaziz University in Jeddah, Saudi Arabia (reference no. 554-22). All participants were required to provide consent for their participation at the beginning of the online questionnaire.

### 2.2 Participants and recruitment

The study inclusion criteria were being male or female, aged 18 years or older, and living in Saudi Arabia. The questionnaire was developed using Google Forms. A direct link to the questionnaire was then shared on social media platforms, including WhatsApp and X.

### 2.3 Study questionnaire

The questionnaire was adapted from previously used surveys (16, 24, 25). Several modifications were made to the original questionnaires, including rewording, adding options, and combining some questions. The questionnaire was developed in English and then translated into Arabic using the Brislin backtranslation method (26, 27). For pre-testing, the questionnaire was reviewed by 5 experts in nutrition (4 PhD holders and one MSc holder) and one medical doctor (a gastroenterologist). The reviewers were asked about the clarity of the questionnaire instructions, questionnaire design, navigation difficulty, and ease of understanding the questions and potential answers. A number of questions and potential answers were then edited based on the comments from the reviewers. The final version of the questionnaire consisted of 4 main sections and required approximately 10 minutes to complete. The main study aims, inclusion criteria, estimated time needed for questionnaire completion, and confidentiality of collected data were all provided at the beginning of the questionnaire.

In the first section, data regarding the sociodemographic and background characteristics of the participants were collected. This included age, gender, marital status, level of education, current work status, income, field of study, details of any chronic diseases, physical activity (28), and smoking habits. Height in centimeters and weight in kilograms(self-reported) were also collected, which were used to calculate body mass index (BMI) (29).

In the second section, the awareness of participants about probiotics and prebiotics was evaluated. Participants were asked if they were familiar with the concept of prebiotics and probiotics and then prompted to rate their level of awareness using a scale ranging from one (indicating no awareness at all) to 10 (indicating maximum awareness). Using quartiles, the scores were classified into 3 categories, namely, low awareness (below the second quartile (0-50^th^ percentile; score = 1-3), moderate awareness (between the second and third quartiles (50–75^th^ percentile; score = 4-7), and high awareness (above the third quartile (>75th percentile; score= score = 8-10). In addition, participants were asked to indicate the first thing that came to their mind when hearing the terms “prebiotics” and “probiotics”.

In the third section, the knowledge of participants about probiotics and prebiotics was examined. Four questions related to probiotics and prebiotics were asked in this section, including their definitions and natural sources. Correct answers were then scored as “1”, while incorrect or “don’t know” answers were scored as “0”. The total score was then calculated for each participant. Participants scoring (0-50^th^ percentile; score = 1) were considered as having low knowledge, those scoring between (50–75^th^ percentile; score = 2-3) were considered as having moderate knowledge, and those scoring (>75th percentile; score= score = 4) were considered as having high knowledge.

In the fourth section, the beliefs of participants regarding the benefits of prebiotic and probiotic consumption were assessed. Participants were asked to predict the benefits of prebiotic and probiotic consumption on several health conditions, including digestion and gut health, immune system support, nutrient absorption, bodily detoxification, stress management, constipation, diarrhea, heart health, overweight/obesity, and mental health/stress. For each condition, they were asked to select one of the following options: “beneficial”, “not beneficial”, or “I don’t know”.

### 2.4 Sample size calculation

The study sample size was calculated using the Raosoft software based on the number of adults living in Saudi Arabia (General Authority for Statistics 2019) (30). With a margin of error of 5%, a confidence level of 95%, and a response distribution of 50%, the recruitment of a minimum of 385 participants was found to be necessary.

### 2.5 Statistical analysis

Statistical analysis was performed using the SPSS software program (Version 28, SPSS Inc., Chicago, IL, USA). Data were expressed as numbers and percentages. The chi-square test was used to investigate the associations between categorical variables. A p value of <0.05 was considered to represent statistical significance.

## 3 Results

### 3.1 Participant demographics and background

**Table 1** summarizes the sociodemographic characteristics of the study population. A total of 1,306 participants completed the survey. Among the study population, approximately half (48.8%) of the participants were aged 18–24 years, and 69.3% were females. Approximately three-fifths (58.8%) of the participants were single, and 56.8% were educated to university level. The dominant income categories reported were <2,000 Saudi riyals (<533 USD) per month (29.6%) and >10,000 Saudi riyals (>2665 USD) per month (26.1%). Almost half (45.7%) of the participants reported their field of study as scientific. Less than 20% of participants reported a history of chronic diseases (18.7%), and being a current smoker (11.0%). Calculation of BMI values indicated that 45.8% of participants were either overweight or obese, and only 23.4% of the participants classified themselves as very active. The 3 most commonly reported sources of dietary information were media (TV, radio) (44.8%), friends/peers/colleagues (44.7%), and organizations (41.1%).

**Table 1:** Sociodemographic and background characteristics of the participants (*n*=1,306).

Variable	Frequency (*n*)	Percentage (%)
Age (years)		
18–24	636	48.8
25–39	353	27.0
40–59	272	20.8
≥60	45	3.4
Gender		
Male	401	30.7
Female	905	69.3
Marital status		
Single	768	58.8
Married	501	38.4
Divorced	26	2.0
Widowed	11	0.8
Educational level		
Less than high school	38	2.9
High school	398	30.5
University	742	56.8
Postgraduate	128	9.8
Work status		
Student	553	42.3
Employed	389	29.8
Unemployed	213	16.3
Retired	90	6.9
Business/trading	61	4.7
Income, Saudi riyals per month (US dollars)		
No income	236	18.1
<2,000 (<533 USD)	387	29.6
2,000–4,000 (533- 1066 USD)	149	11.4
4,001–7,000 (1067-1865 USD)	102	7.8
7,001–10,000 (1866- 2664 USD)	91	7.0
>10,000 (>2665 USD)	341	26.1
Field of study		
Medical	251	19.2
Scientific	597	45.7
Literature	351	26.9
No specific field	107	8.2
History of chronic diseases		
No	1,062	81.3
Yes	244	18.7
Physical activity ^a^		
Fairly inactive	553	42.3
Moderately active	448	34.3
Very active	305	23.4
Smoking		
No	1,108	84.8
Yes	144	11.0
Ex-smoker	54	4.1
BMI (*n*=1,294) ^b^		
Underweight	128	9.9
Normal	573	44.3
Overweight	353	27.3
Obese	240	18.5
Source of dietary information		
Family members	300	23.0
Friends/peers/colleagues	583	44.7
Books/magazines	406	31.1
Internet/website	70	5.4
Media (TV, radio)	585	44.8
Social media	114	8.8
Healthcare professionals	293	22.5
Organizations	536	41.1

a Fairly inactive (walking only); moderately active (occasionally take exercise, that rise heart rate, less than 3 times per week); very active (regularly take exercise, that rise heart rate, less than 3 times per week or more)

b Calculated based on self-reported weight and height.

### 3.2 Awareness of probiotics and prebiotics

**Figure 1:**
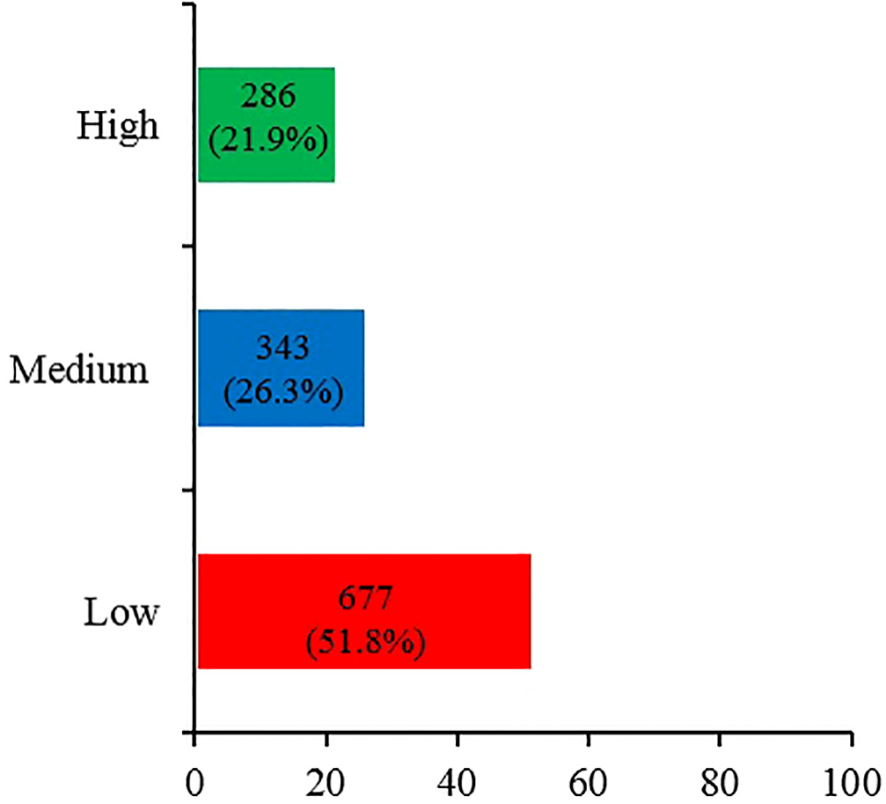
Awareness of probiotics and prebiotics among the participants (n=1,306).

**Figure 1** shows the levels of awareness regarding probiotics and prebiotics reported by the participants. A high level of awareness was reported by only 21.9% of participants, while more than half (51.8%) of participants reported a low level of awareness.

### 3.3 Knowledge about probiotics and prebiotics

**Table 2** presents the knowledge regarding probiotics and prebiotics. The majority (78.4%) of the participants recognized that yogurt is one food that may be a natural source of probiotics, while 68.8% knew correctly that fruit and vegetables and whole grains are natural sources of prebiotics. Almost three-quarters (73.9%) of participants knew what probiotics are, while only 57.2% knew what prebiotics are. Overall, 37.5% displayed a high level of knowledge about prebiotics and probiotics, whereas 15.5% had a low level of knowledge.

**Table 2:** Knowledge and awareness levels about probiotics and prebiotics among the participants (*n*=1,306).

A. Knowledge level					
Question		Correct answer	Low (<50th percentile)	Moderate (50–75th percentile)	High (>75th percentile)
		*n* (%)	*n* (%)	*n* (%)	*n* (%)
Q1	What are probiotics?	965 (73.9)			
Q2	What are prebiotics?	747 (57.2)			
Q3	Which of the following foods may be a natural source of probiotics?	1,024 (78.4)	203 (15.5)	613 (46.9)	490 (37.5)
Q4	Which of the following foods may be a natural source of prebiotics?	898 (68.8)			
B. Awareness level					
Are you aware of the concept of pro/prebiotics?			677 (51.8%)	343 (26.3%)	286 (21.9%)

*n* (%): Data are presented as number and percentage.

As shown in **Table 3**, the highest rate of a high level of knowledge was observed among participants aged between 25 and 39 years (43.6%), whereas the lowest rate of a high level of knowledge was found for those aged between 18 and 24 years (32.9%) (*p*=0.001). Postgraduates displayed a higher rate of a high level of knowledge than individuals educated only to high school level (48.5% vs. 31.2%, *p*=0.005). Unemployed persons exhibited the highest rate of a high level of knowledge (43.6%), whereas students displayed the lowest rate (32.5%) (*p*=0.017). Participants whose field of study was medical had the highest rate of a high level of knowledge (57.3%), while those without a specific field of study had the lowest rate (21.5%) (*p*<0.001). Participants with a history of chronic diseases were more knowledgeable about prebiotics and probiotics compared with their peers (44.7% vs. 35.9%, *p*=0.029).

**Table 3:** Factors associated with the level of knowledge of the participants about probiotics and prebiotics (*n*=1,306).

	Level of probiotic and prebiotic knowledge			*p* value^†^
	Low 203 (15.5%)*	Moderate 613 (46.9%)*	High 490 (37.5%)*	
Age (years)				
18–24 (*n*=636)	125 (19.7)	302 (47.4)	209 (32.9)	0.001
25–39 (*n*=353)	43 (12.2)	156 (44.2)	154 (43.6)	
40–59 (*n*=272)	31 (11.4)	132 (48.5)	109 (40.1)	
≥60 (*n*=45)	4 (8.9)	23 (51.1)	18 (40.0)	
Gender				
Male (*n*=401)	74 (18.5)	188 (46.8)	139 (34.7)	0.110
Female (*n*=905)	129 (14.3)	425 (46.9)	351 (38.8)	
Marital status				
Single (*n*=768)	144 (18.8)	358 (46.6)	266 (34.6)	0.002
Married (*n*=501)	56 (11.2)	242 (48.3)	203 (40.5)	
Divorced (*n*=26)	2 (7.7)	9 (34.6)	15 (57.7)	
Widowed (*n*=11)	1 (9.1)	4 (36.4)	6 (54.5)	
Educational level				
Less than high school (*n*=38)	5 (13.2)	18 (47.3)	15 (39.5)	0.005
High school (*n*=398)	74 (18.6)	200 (50.2)	124 (31.2)	
University (*n*=742)	115 (15.5)	338 (45.6)	289 (38.9)	
Postgraduate (*n*=128)	9 (7.0)	57 (44.5)	62 (48.5)	
Work status				
Student (*n*=553)	101 (18.3)	272 (49.2)	180 (32.5)	0.017
Employed (*n*=389)	57 (14.7)	169 (43.4)	163 (41.9)	
Unemployed (*n*=213)	30 (14.1)	90 (42.3)	93 (43.6)	
Retired (*n*=90)	8 (8.9)	49 (54.4)	33 (36.7)	
Business/trading (*n*=61)	7 (11.5)	33 (54.1)	21 (34.4)	
Income, Saudi riyals per month (US dollars)				
No income (*n*=236)	40 (16.9)	123 (52.2)	73 (30.9)	0.088
<2,000 (<533 USD) (*n*=387)	72 (18.6)	176 (45.5)	139 (35.9)	
2,000–4,000 (533- 1066 USD) (*n*=149)	23 (15.4)	62 (41.6)	64 (43.0)	
4,001–7,000 (1067-1865 USD) (*n*=102)	19 (18.6)	44 (43.2)	39 (38.2)	
7,001–10,000 (1866- 2664 USD) (*n*=91)	11 (12.1)	42 (46.1)	38 (41.8)	
>10,000 (>2665 USD) (*n*=341)	38 (11.1)	166 (48.7)	137 (40.2)	
Field of study				
Medical (*n*=251)	17 (6.8)	90 (35.9)	144 (57.3)	<0.001
Scientific (*n*=597)	104 (17.4)	280 (46.9)	213 (35.7)	
Literature (*n*=351)	62 (17.7)	179 (51.0)	110 (31.3)	
No specific field (*n*=107)	20 (18.7)	64 (59.8)	23 (21.5)	
History of chronic diseases				
No (*n*=1,062)	173 (16.3)	508 (47.8)	381 (35.9)	0.029
Yes (*n*=224)	30 (12.3)	105 (43.0)	109 (44.7)	
Smoking				
No (*n*=1,108)	170 (15.3)	518 (46.8)	420 (37.9)	0.390
Yes (*n*=144)	22 (15.3)	75 (52.1)	47 (32.6)	
Ex-smoker (*n*=54)	11 (20.4)	20 (37.0)	23 (42.6)	
BMI (*n*=1,294)				
Underweight (*n*=128)	25 (19.5)	61 (47.7)	42 (32.8)	0.293
Normal (*n*=573)	101 (17.6)	265 (46.3)	207 (36.1)	
Overweight (*n*=353)	44 (12.5)	168 (47.6)	141 (39.9)	
Obese (*n*=240)	33 (13.8)	114 (47.4)	93 (38.8)	

* *n* (%): Data are presented as number and percentage. ^†^ *p* values were calculated using chi-square tests between the selected variables.

### 3.4 Common terms associated with probiotics and prebiotics

**Figure 2:**
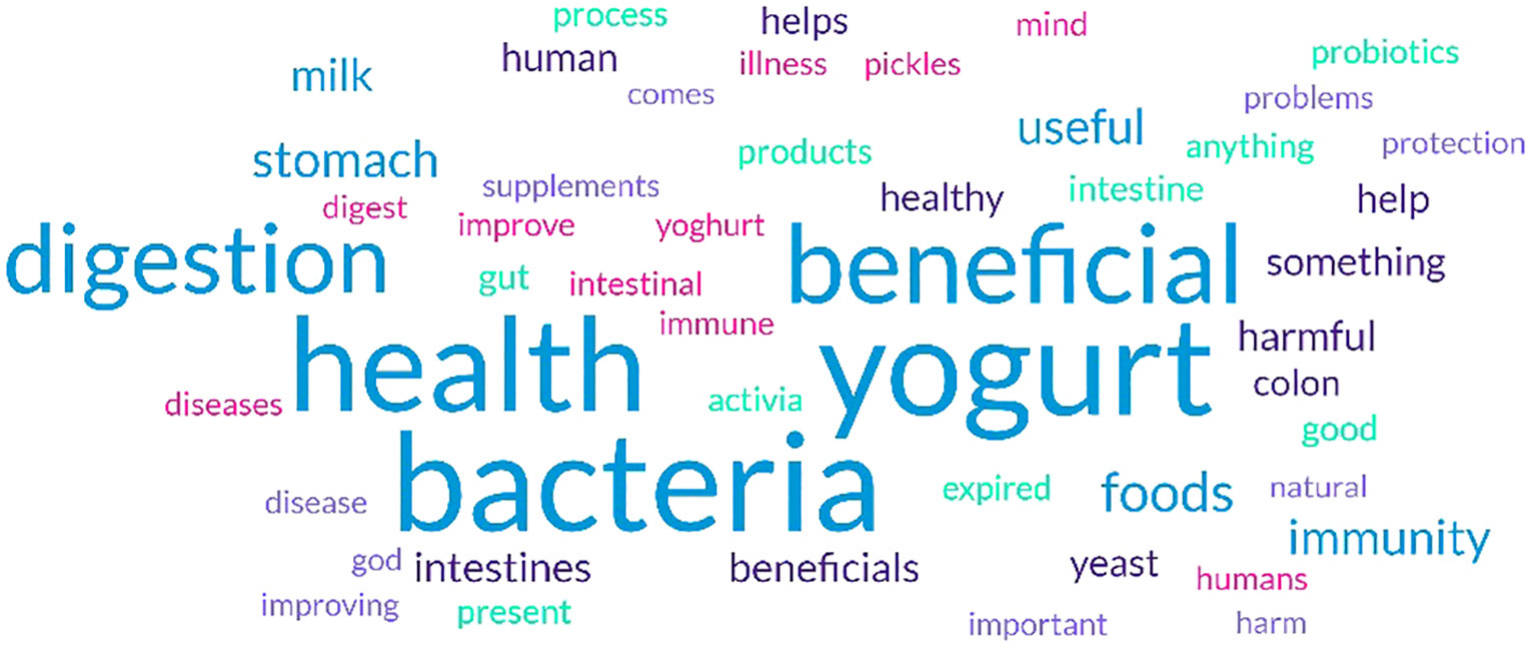
Word cloud illustrating the frequency of responses in the word association exercise.

As shown in **Figure 2** and **Table 4**, the 5 most frequent words were associated with probiotics and prebiotics “yogurt”, “bacteria”, “health”, “beneficial”, and “digestion”. The 10 most frequent responses accounted for almost 91% for all responses, while 578 responses were given less than 30 times (9% of all responses).

**Table 4:** Top 10 most frequently given unique responses in the word association exercise.

Rank	Frequency		Word
	*n*	%	
1	223	17	Yogurt
2	222	17	Bacteria
3	207	16	Health
4	166	13	Beneficial
5	139	11	Digestion
6	55	4	Foods
7	46	4	Stomach
8	43	3	Immunity
9	42	3	Useful
10	43	3	Milk

### 3.5 Perceived beliefs about the beneficial effects of probiotics and prebiotics

As shown in **Table 5**, the majority of participants believed in the beneficial effects of probiotics and prebiotics in terms of overall digestion/gut health (84.1%) and supporting the immune system (72.5%). Over half believed probiotics/prebiotics were also beneficial for absorption of nutrients (63.5%), detoxifying the body (60.8%), constipation (59.2%) and diarrhea (52.0%). However, less than half of participants believed in the beneficial effects of prebiotics and probiotics with respect to overweight/obesity (42.3%), stress management (35.0%), mental health/stress (29.2%), and heart health (28.7%). While, 14.9% of participants did not know the beneficial effects of probiotics and prebiotics on overall digestion/gut health, supporting the immune system (22.8%), absorption of nutrients (31.4%), detoxifying the body (32.2%), stress management (50.0%), constipation (31.6%), diarrhea (37.1%), heart health (55.1%), overweight/obesity (44.1%), mental health/stress (52.7%).

**Table 5:** Beliefs of participants regarding the beneficial effects of probiotics and prebiotics (*n*=1,306).

Health condition	Beneficial *n* (%)	Not beneficial *n* (%)	Don’t know *n* (%)
Overall digestion/gut health	1,099 (84.1)	13 (1.0)	194 (14.9)
Supporting the immune system	946 (72.5)	62 (4.7)	298 (22.8)
Absorption of nutrients	829 (63.5)	67 (5.1)	410 (31.4)
Detoxifying the body	794 (60.8)	92 (7.0)	420 (32.2)
Stress management	457 (35.0)	196 (15.0)	653 (50.0)
Constipation	773 (59.2)	120 (9.2)	413 (31.6)
Diarrhea	678 (52.0)	143 (10.9)	485 (37.1)
Heart health	375 (28.7)	212 (16.2)	719 (55.1)
Overweight/obesity	553 (42.3)	178 (13.6)	575 (44.1)
Mental health/stress	381 (29.2)	237 (18.1)	688 (52.7)

*n* (%): Data are presented as number and percentage.

## 4 Discussion

This study aimed to address awareness, knowledge, and beliefs about probiotics and prebiotics among Saudi adults. Despite growing interest in probiotics and prebiotics in the management and treatment of chronic diseases, understanding people’s knowledge and beliefs is crucial for attempts to promote healthier lifestyles (1, 6, 8, 31–34). Until now no studies have been performed to evaluate these factors among the general public in Saudi Arabia, although a few studies have conducted to measure this knowledge among college students and healthcare professionals (21–23). The results of this study could contribute to a broader understanding of public attitudes toward probiotics and prebiotics and provide useful information to researchers, policymakers, and companies to support future research and strategies. This information is also expected to prove useful for address the needs and preferences of consumers to improve their overall health.

The study population was composed of a diverse demographic profile, with approximately half of participants aged 18–24 years (48.8%) and overweight or obese (45.8%). The majority of participants reported that they relied on a mixture of sources to obtain dietary information, including media, friends/peers/colleagues, and organizations. Approximately half (51.8%) of participants reported themselves as having low awareness of the terms “prebiotic” and “probiotic”, 26.3% reported themselves as having moderate awareness, and only 21.9% of participants reported themselves as having high awareness. This is consistent with previous study (Al-Qassim, 2019) as 26% of the Saudi participants reported that they had heard of and are aware of probiotics terms (13). Although there are five years between the two studies, the current study did not reveal an improvement in public awareness towards probiotics. This could be due to insufficient or varied educational health messages that make it difficult for the general public to recognize and be aware of the terms “prebiotic” and “probiotic”. In addition, this also could be due to regional disparities. The Al-Qassim study (2019) was conducted in one city, while the data of the current study were collected from all regions. At the international level, this study is also aligned with a study by Khalesi et al. (2021) (16), who reported a low overall awareness of gut flora, prebiotics, and probiotics among Australian adults. However, assessment of the knowledge of the participants in this study using a scoring system based on 4 questions revealed a different picture. The majority of participants (84.4%) displayed a level of knowledge that was either high (37.5%) or moderate (46.9%), while only 15.5% exhibited a low level of knowledge. Approximately four-fifths (78.4%) of participants correctly recognized that yogurt is a natural source of probiotics, and over two-thirds (68.8%) knew that fruits, vegetables, and whole grains are natural sources of prebiotics. These results show that the majority of participants had a good understanding of the sources of prebiotics and probiotics. However, fewer participants could correctly define prebiotics (57.2%) and probiotics (73.9%). This suggests that the definition and source of prebiotics were not as commonly recognized by the study population. These findings indicate a discrepancy between the self-assessed awareness of participants and their demonstrated high knowledge, with many participants rating their awareness as low despite exhibiting moderate to high levels of knowledge. This could stem from factors such as political ideology, previous negative experiences, or concerns regarding the credibility of information (35). For example, political ideology might influence how prebiotics and probiotics information is accepted or rejected based on alignment with personal or group beliefs. Moreover, even if individuals are aware of the potential health benefits of prebiotics and probiotics, previous negative experiences with health products might lead to uncertainty or hesitancy to entirely embrace prebiotics and probiotics. In addition, concerns about information credibility may influence awareness; with lots of varying information available, individuals might struggle to discern what is reliable, leading to a superficial understanding rather than a deep awareness of how prebiotics and probiotics can be effectively used. Further studies are needed to understand the psychological and contextual factors that may affect self-assessments of awareness in relation to actual knowledge levels.

Understanding the factors associated with the variation in knowledge levels and the role of sociodemographic characteristics may assist the design of targeted educational interventions aimed at bridging this knowledge gap. One of the most important factors underlying differences in knowledge levels is age. The highest levels of knowledge were observed among participants aged 25–39 years, while the lowest levels were found for participants aged 18–24 years. The obtained results are not comparable to other studies in which students were found to display fair to good knowledge about probiotics, which may be because these studies focused only on medical students (36–38). The results may suggest that younger participants had fewer opportunities or less motivation to learn about probiotics and prebiotics.

Educational level is another factor, with postgraduate degree holders exhibiting a higher rate of a high level of knowledge compared with individuals possessing only a high school degree (25). Participants whose field of study was medical also displayed a higher rate of a high level of knowledge than participants without a specific field of study. This may be because medical education has a high likelihood of providing comprehensive information regarding the roles of prebiotics and probiotics. In addition, participants with a history of chronic diseases were found to be more knowledgeable about prebiotics and probiotics than their healthier counterparts. This may be attributable to their greater exposure to medical services (39).

A survey carried out in Turkey found that 87.0% of adult consumers were familiar with probiotics, whereas 62.2% were knowledgeable about prebiotics (40). A study of Romanian consumers revealed that 74% were aware of prebiotics, while 25% were unfamiliar with the concept (24). A study conducted in Australia examined the level of understanding and opinions about gut health, prebiotics, and probiotics, where 66% of the participants correctly identified the term “gut flora” and 76.6% correctly identified the term “probiotics” (16). On the other hand, only 35.3% recognized the definition of “prebiotics” and more than half (58.6%) were not familiar with the term. The majority of participants (77.7%) accurately identified yogurt as a natural source of beneficial bacteria. Finally, research conducted in the United Arab Emirates indicated a lack of awareness about the term “probiotics” and its meaning, with over 75% of participants not knowing the distinction between prebiotics and probiotics and misunderstanding how they are used (41). Overall, previous research indicates that consumers are generally well informed about probiotics, but their knowledge about prebiotics is less extensive (16, 24, 40, 41) Thus, more education and awareness initiatives are needed to enhance public understanding of these crucial concepts in gut health.

In accordance with their good understanding of prebiotics and probiotics, the participants generated a variety of words that are widely associated with these terms when they were asked “What is the first thing that comes into your mind when hearing prebiotics and probiotics?”. The words tended to focus on the health benefits and digestive effects of prebiotics and probiotics, such as “yogurt”, “bacteria”, “health”, “beneficial”, and “digestion”. The majority of participants immediately think of ‘yogurt’, which reflects the association between probiotics and fermented dairy products. Moreover, the word ‘bacteria’ indicates an understanding that probiotics involve live microorganisms that are beneficial for health. ‘Health’ was indicated, suggesting that participants realize that probiotics contribute to general well-being. Lastly, the references to ‘beneficial’ and ‘digestion’ highlight that people specifically associate these terms with their impact on digestive health. This may be attributable to the prevalent use of a mixture of sources such as media, friends/peers/colleagues, and organizations to obtain dietary information.

The results showed that the majority of participants believed that probiotics and prebiotics exert beneficial effects on digestion and gut health (84.1%) and provide support to the immune system (72.5%). This suggests that there exists widespread awareness of the benefits of prebiotics and probiotics for gut health and good recognition of the link between gut health and immune functionality. However, less than half (42.3%) of participants believed that prebiotics and probiotics could contribute to weight management, which indicates that the relationship between gut health and weight management is not well understood by the general public. In addition, the proportions of participants who believed that probiotics and prebiotics were beneficial for stress management (35.0%), mental health/stress (29.2%), and heart health (28.7%) were even lower. Only one previous study found significant associations between participants understanding what probiotics are, knowing the benefit of consuming probiotics on improving immune function, and frequency of probiotic consumption (42).

Like most cross-sectional studies, this study has several limitations. Therefore, the reported findings need to be interpreted with caution. First, this cross-sectional analysis, combined with self-reported methods, only addresses associations between variables but cannot prove causality and may introduce bias associated with recalling and question interpretation. Second, the generalizability of the study is limited by the fact that the sociodemographic data may not reflect the entire Saudi population. Convenience sampling and social media platforms were used to disseminate the electronic questionnaire, which may have introduced some minor bias. Third, the current study did not evaluate participants’ awareness and knowledge of differences in effectiveness of probiotics and prebiotics due to differences in strain-specificity and product types. Studies have shown that not all probiotics and prebiotics are equally effective, and strain-specificity is extremely important when trying to choose an appropriate probiotic product (43). In addition, the survey did not ask participants about their preferred probiotic and prebiotic sources and did not determine whether participants understood the varying effectiveness of different probiotic and prebiotic types (44). However, despite the limitations of this study, the significant large sample size increased the statistical power to detect the association between the study variables and the level of knowledge of the participants about probiotics and prebiotics. In addition, the use convenience method with an online-administrated facilitated faster data collection and allowed access to diverse range participants across various locations, making the findings more applicable to a border population. Future studies need to capture the study limitations in order to provide a comprehensive understanding to recognize the distinctions of selecting effective probiotic and prebiotic products. Selecting the correct product is crucial for optimal health outcomes. Development of educational materials are necessary to allow informed decision-making, as well as to guide industry and product developers to design formulations that meet the needs of individuals and the market.

## 5 Conclusion

This study provides valuable insights into the current levels of awareness, knowledge, and beliefs regarding probiotics and prebiotics among the Saudi population. The obtained findings indicate sufficient knowledge levels about prebiotics and probiotics among a population sample of Saudi adults. However, enhanced educational efforts and optimized strategies for promoting a comprehensive awareness and understanding of prebiotics and probiotics are recommended. The findings of this study may serve as a foundation for researchers, policymakers, and industry leaders supporting future studies and strategies, as well as efforts to address the needs and preferences of consumers and help them improve their overall gut health.

## Data Availability

The raw data supporting the conclusions of this article will be made available by the authors, without undue reservation.
