# Supplementary material for: Awareness, knowledge, and beliefs about probiotics and prebiotics among Saudi adults: a cross-sectional study
Source: Front Immunol. 2024 Oct 24;15:1464622. doi: 10.3389/fimmu.2024.1464622 (PMC11540690; doi:10.3389/fimmu.2024.1464622)
Supplement: Supplementary file 1 [file Table1.docx]

**Supplemental File**

| **Section 1: Sociodemographic and background section:** | |
| --- | --- |
| 1. How old are you?   ……………. (years) | 1. Gender:  - Male - Female |
| 1. What is your marital status?  - Single - Married - Divorced - Widowed | 1. What is your highest level of education?  - Less than high school - High school or equivalent - Bachelor's degree - Master’s degree or higher |
| 1. What is your current work status?  - Student - Employed - Unemployed - Retired - Business/trading | 1. What range of income do you have? (SR)  - No income - Below 2000SAR - 2000 - 4000SAR - 4,001–7,000 SAR - 7,001–10,000 SAR - Over 10000SAR |
| 1. What is your specialty?  - Medicine or medical sciences - Science - Literature - No specific field (high school students/education was not completed) | 1. Have you been diagnosed by your doctor with any of the following?  - Yes, I have been diagnosed - No, I haven't been diagnosed |
| 1. Do you smoke?  - Yes - No - I am an ex-smoker | 1. What is your height?   ……………. cm |
| 1. How much do you weigh?   ……………. kg | 1. From where do you seek information about your diet?  - Family members - Friends/peers/colleagues - Books/Magazines - Internet website - Media (TV, radio) - Social media (Facebook, Twitter, Instagram, etc) - Health care professionals (doctor, nurse, dietitian, etc) - Organizations (MOH, WHO, government, associations) |
| 1. Which region do you reside in?  - Central region - Northern region - Southern region - Eastern region - Western region | 1. What city do you live in? |
| **Section 2: Questions related to awareness** | |
| 1. Are you aware of the concept of pro/prebiotics? (1 means you do not know anything about it, and 10 means you know a great deal of information)  \| **1** \| **2** \| **3** \| **4** \| **5** \| **6** \| **7** \| **8** \| **9** \| **10** \| \| --- \| --- \| --- \| --- \| --- \| --- \| --- \| --- \| --- \| --- \| | 1. What was the first thing that came into your mind when hearing pro/prebiotics?   …………………../……………../……………… |
| **Section 3: Questions related to knowledge** | |
| 1. To the best of your knowledge, what are **’probiotics’**?  - Live microorganisms that when digested can be beneficial to humans - A vitamin supplements - Antibiotics prescribed to treat infections caused by bacteria - Don’t know - Never heard | 1. To the best of your knowledge, what are **’prebiotics’**?  - Dietary fiber that good bacteria need to survive - A vitamin supplements - Antibiotics prescribed to treat infections caused by bacteria - Don’t know - Never heard |
| 1. To the best of your knowledge, which one of the following foods may be a natural source of **probiotics**?  - Yoghurt - Fruit and vegetables and whole grains - Foods cannot be a natural source of probiotics - Don’t know - Never heard | |
| 1. To the best of your knowledge, which one of the following foods may be a natural source of **prebiotics**?  - Yoghurt - Fruit and vegetables and whole grains - Foods cannot be a natural source of probiotics - Don’t know - Never heard | |
| **Section 4: Questions related to believe** | |
| 1. Do you believe that pro/probiotics is beneficial for:  \|  \| **Pro/prebiotics** \| \| \| \| --- \| --- \| --- \| --- \| \| Beneficial \| Not beneficial \| I do not know \| \| **Overall digestion/gut health** \|  \|  \|  \| \| **Support the immune system** \|  \|  \|  \| \| **Absorption of nutrients** \|  \|  \|  \| \| **Detoxify the body** \|  \|  \|  \| \| **Stress management** \|  \|  \|  \| \| **Constipation** \|  \|  \|  \| \| **Diarrhea** \|  \|  \|  \| \| **Heart health** \|  \|  \|  \| \| **Overweight/obesity** \|  \|  \|  \| \| **Mental health/stress** \|  \|  \|  \| | |

**Thank you for your time with us**
